# Effect of Squid Cartilage Chitosan Molecular Structure on the Properties of Its Monofilament as an Absorbable Surgical Suture

**DOI:** 10.3390/polym14071306

**Published:** 2022-03-24

**Authors:** Yongxin Tan, Muhammad Shahid Riaz Rajoka, Zekai Ke, Hafiza Mahreen Mehwish, Wenjing Deng, Jiaying Li, Wenqian Qin, Liqing Zhao, Yiguang Wu

**Affiliations:** 1 Department of Food Science and Engineering, College of Chemistry and Chemical Engineering, Shenzhen University, Shenzhen 518060, China; tanyonxin@126.com (Y.T.); shahidrajoka@yahoo.com (M.S.R.R.); 15875513174@163.com (W.D.); 2060221012@email.szu.edu.cn (J.L.); 2060221011@email.szu.edu.cn (W.Q.); 2 Department of Pharmacy, Health Science Center, Shenzhen University, Shenzhen 518060, China; mahreen.mehwish@yahoo.com; 3 Food and Feed Immunology Group, Graduate School of Agricultural Science, Tohoku University, Sendai 980-8572, Japan; 4 Department of Orthopaedics, Shenzhen University General Hospital, Shenzhen University Clinical Medical Academy, Shenzhen 518060, China; zekaike@szu.edu.cn

**Keywords:** squid cartilage chitosan, absorbable surgical suture, wet spinning, degree of deacetylation, viscosity average molecular weight

## Abstract

Suture is an important part of surgery, and wounds closing after surgery remains a challenge for postoperative care. Currently, silk, linen fiber, and cotton are available in the market as non-absorbable suture biomaterials. So, there is an urgent need to develop a novel suture with advantageous characteristics compared to the ones available on the market. In present study, a series of ultra-high molecular weight chitosan with different DD and M_V_ were prepared from squid cartilage by alkaline treatment and ultrasonic degradation. The corresponding chitosan monofilaments were prepared by a wet spinning process and were characterized as sutures. The effects of the DD and M_V_ of chitosan on the properties of its monofilament were studied, including surface morphology, mechanical property, swelling ratio, ash content, in vitro enzymatic degradation, and in vitro cytotoxicity. According to the results, AS-85 was chosen to be the best suitable as an absorbable surgical suture, which was spun from squid cartilage chitosan with DD~85% and M_V_~1.2 × 10^6^. The outcome of the present study might derive tremendous possibilities for the utilization of squid cartilage β-chitin for biomedical applications.

## 1. Introduction

Sutures are the medical devices which were used for wound healing, to repair damaged tissues, and to obligate the blood vessels [1,2]. So, the suture materials have a wide range of applications in the field of surgery, and the demand for suture grows at the rate of millions of dollars annually. The basic purpose of a suture is to stabilize wounds as well as to promote wound healing. That is why the biocompatibility and mechanical properties are vital for an ideal suture biomaterial [3,4]. According to its origin, the suture can be divided into natural sutures (silk, cotton, and linen fiber) and synthetic sutures (nylon and polyester). Furthermore, according to degradation properties, sutures can be divided into non absorbable and absorbable sutures [5,6].

After wound healing, the non-absorbable sutures need to be removed from the body, which might cause further injury, thereby making the procedure more complicated as well as painful for the patients [7]. Additionally, the removal of sutures in pediatric patients and difficulty of accessing anatomical areas are clinically challenging [8]. On the other hand, absorbable sutures do not need to be removed after surgery. Hence, they help reduce the incidence of second injury as well as reduce the patient’s pain [2,9].

An absorbable surgical suture is one of the most important in the development of polymer materials [10]. Compared with non-absorbable sutures, an absorbable suture is biodegradable, avoiding the risk of secondary operation [11]. Absorbable surgical sutures include natural fibers and synthetic polymers. The former includes catgut [12], collagen [13], and chitin fiber [14]. The latter includes polyglycolic acid (PGA) [15], poly (p-dioxanone) (PDS) [16], poly (lactic-co-glycolic acid) (PGLA) [17], poly (trimethylene carbonate) (PTMC) [18], and polycaprolactone (PCL) [19].

The ideal absorbable suture should be flexible and have high knotted strength; it should be convenient for operation with a strong ability to remain knotted, maintaining a certain strength in the body for a certain time and then being absorbed after the wound heals. It should have good biocompatibility, with no inflammation due to foreign body reaction. Finally, it should be stable, reliable, thoroughly sterilizable, and convenient for storage [4,20,21]. Moreover, the development of the ideal absorbable surgical suture needs to consider practical factors, so it is difficult to prepare a suture that meets all clinical needs [22,23]. Different types of wound tissue have different requirements for suturing, such as fascia or tendon tissue, which need weeks or even months to repair, so a suture with a long degradation cycle is needed. Wounds in the muscles and epithelial tissue can be healed in a few days, so a suture with a short degradation cycle is needed [24].

Chitosan is the only alkaline polysaccharide found in nature, which has abundant and unique properties, including good biocompatibility, degradability, cell adhesion, hemostasis and antibacterial properties, and wound-healing effects [25,26,27]. Moreover, the amino sugars produced by chitosan degradation can be completely absorbed by the human body. All of these criteria make chitosan a viable candidate for absorbable surgical sutures [28,29].

In this paper, β-chitin extracted from squid cartilage was used as the raw material, and then a series of ultra-high molecular weight chitosan with different deacetylation degree (DD) and viscosity average molecular weight (M_V_) were prepared by the method of alkaline deacetylation and ultrasonic degradation. The corresponding chitosan monofilaments were prepared by a wet spinning process and were characterized as sutures. The effects of the DD and M_V_ of chitosan on the properties of its monofilament were studied. This study aimed to carry out the basic research of suture selection in line with clinical needs.

## 2. Experimental

### 2.1. Materials

β-chitin was supplied by Jiangxi Goldenbrilliance Medical Products Co., Ltd., China. NaOH (AR), acetic acid (AR), HCl (AR), and ethanol (AR) were purchased from Aladdin (Shanghai, China). Deionized water was used throughout the experiments.

### 2.2. Preparation and Characterization of Chitosan

Chitosan with high M_V_ were prepared by milder alkaline treatment. β-chitin or chitosan powder was dispersed in an NaOH (0.1 M) solution with a solid-liquid ratio of 1:11 (g/mL) under stirring. Deacetylation was processed at the appropriate temperature. After reaction, the mixture was filtered and the solid was washed with deionized water until neutral and was then dried in an oven at 80 °C.

Chitosan with different M_V_ were prepared by ultrasonic degradation. Chitosan with ultra-high M_V_ was dispersed in a 1.0% (*w/w*) of HCl (0.1 M) solution with stirring until it dissolved at room temperature, then the solution was put into an ultrasonic cleaner at 60 °C. After degradation, the solution was adjusted to pH 7 by the addition of 1.0 mol/L of NaOH solution until complete precipitating. The mixture was filtered and the solid was washed with deionized water and ethanol and was then dried in an oven at 80 °C.

The DD of chitosan was determined by elemental analysis (Vario micro cube Element Analyzer, Elementar Analysis System GmbH, Langenselbold, Germany). The M_V_ of chitosan was examined by the dilute solution viscosity method.

### 2.3. Wet Spinning to Produce Chitosan Monofilaments

The chitosan monofilament was wet spun in a wet spinning machine. Briefly, 3.0 g of chitosan was dissolved in 100 mL, 2.0% (*v/v*) of acetic acid solution at room temperature. The chitosan acetate solution was filtered and defoamed before wet spinning. The solution was loaded in a syringe of the wet spinning machine and squeezed at an extrusion pressure of 1.4 MPa into the coagulation bath which was comprised of 5.0% (*w/w*) sodium hydroxide and 2.0% (*v/v*) ethanol aqueous solution. Then, the precipitated monofilament was washed to neutrality with deionized water. All monofilaments spun from chitosan with different DD and M_V_ were collected and dried naturally at room temperature on the collector.

### 2.4. Characterization of Chitosan Monofilaments

The appearance morphology of chitosan monofilament was observed by SEM (S3400N (II), Hitachi, Japan) at an accelerating voltage of 10 kV. All samples were treated with gold spray for 80 s before imaging.

The dry or wet monofilament was tied in a knot at about 10 cm and ten specimens were evaluated for each sample. The tensile rate was set at 300 ± 50 mm/min and the test gauge distances were 125~200 mm. Before testing, the wet monofilament was treated by first immersing the dry in 0.1 mol/L of PBS (pH 7.4) for 24 h, and clearing the excess PBS on its surface by filter paper.

The sample was soaked in 0.1 mol/L of PBS (pH 7.4) for 24 h, the excess PBS on its surface was gently absorbed by filter paper, and it was weighed (noted as *W_t_*). Then, it was dried in a vacuum oven at 100 °C for 6 h, and the dry was weighed (noted as *W_d_*). Three specimens were tested for each sample, and the swelling ratio of the chitosan monofilament was calculated as follows:

(1) $$\mathrm{Swelling}\;\mathrm{ratio}=\frac{W_{d}-W_{t}}{W_{t}}\times 100\%$$

The sample was dried in an oven at 120 °C for 2 h and weighed (noted as *W*_0_) after cooling. Then, the sample was cut into pieces and placed into a crucible. The crucible with the dried sample was weighed (noted as *W*_1_). The ash content was determined by fully carbonizing for 3 h and ashing in a muffle furnace at 600 °C for at least 5 h. After cooling, the crucible with ashed sample was weighed (noted as *W*_2_). The ash value was calculated as the following equation:

(2) $$\mathrm{Ash}\%\;(\mathrm{dry}\;\mathrm{basis})=\frac{W_{2}-W_{0}}{W_{1}-W_{0}}\times 100$$

The in vitro enzymatic degradation performance of chitosan monofilament was characterized by the residual ratio, the breaking strength retention (BSR), and the morphological observation. The dried sample was weighed (noted as *W*_0_) and immersed in 50 mL of PBS containing 1 mg/mL of lysozyme (>20,000 U/mg). Then, the sample was incubated at 37 °C with gentle vibration. The fresh lysozyme solution was renewed every three days. The sample was taken out every week and rinsed with deionized water. Then, the sample was dried in a vacuum oven at 100 °C for 2 h and weighed (noted as *W_t_*). The residual ratio was calculated by the following equation:

(3) $$\mathrm{Residual}\;\mathrm{ratio}\%=\frac{W_{t}}{W_{0}}\times 100$$

After the excess PBS on the surface of the chitosan monofilament sample was absorbed by filter paper, it was knotted at the midpoint of the sample. Then, the sample was loaded on the above material testing machine to obtain the tensile strength value. The BSR was calculated by the following formula:

(4) $$\mathrm{BSR}\%=\frac{{\mathrm{TS}}_{t}}{TS_{0}}\times 100$$

*TS_t_* is the tensile strength of the degraded sample at each time point, and *TS_0_* is for the initial.

The degraded sample was dried naturally at room temperature to a constant weight and observed by the above SEM.

### 2.5. In Vitro Cytotoxicity Test of AS-85

An in vitro cytotoxicity test of human skin epithelial cells (HFF-1) after exposure to suture extracts was assessed using the colorimetric MTT assay. Briefly, by comparing the viability of HFF-1 cells cultured with a normal medium to that of the cells cultured with a medium containing suture extracts, AS-85 were selected as the test samples. To prepare the extract, 0.1 g of AS-85 was immersed into 1 mL of Dulbecco’s modified Eagle medium (DMEM) containing 10% (*w/w*) of FBS, 1% (*w/w*) of penicillin and streptomycin at 37 °C for 24 h. Afterwards, the HFF-1 cells were seeded into 96-well plates with a density of 1 × 10^4^ cells/well in DMEM. The medium was replaced 24 h later with a fresh one containing the extracts and the HFF-1 cells were incubated up to another 24 h, 48 h, and 72 h. After each interval, 25 μL of MTT solution (5 mg/mL) was added to each well and incubated for 4 h. After the MTT was removed, 150 μL dimethyl sulfoxide (DMSO) was added and incubated for additional 20 min. The plate was detected by a microplate reader (Thermo Fisher Scientific mμltiscan MK3, Waltham, MA, USA) at a wavelength of 490 nm. The relative cell proliferation rate (RGR) was calculated by the following formula:

(5) $$\mathrm{RGR}=\frac{{\mathrm{OD}}_{test}-{\mathrm{OD}}_{Blank}}{{\mathrm{OD}}_{control}-{\mathrm{OD}}_{Blank}}\times 100$$

*OD_test_, OD_control_* and *OD_blank_* are the OD values for cells cultured in the suture extracts, DMEM, and blank PBS well, respectively.

### 2.6. Statistical Analyses

The results are indicated as mean values ± SD of at least three replicates. The data were expressed as the mean ± SD. The T-test was used to determine the significant differences between the treatment groups by using GraphPad prism 8.0.1.

## 3. Results and Discussion

### 3.1. Structural Parameters of Chitosan and Specifications of Their Monofilaments

The DD and M_V_ of the chitosan prepared under different conditions were shown in Tables S1 and S2. The DD and M_V_ of the chitosan were conditioned by the reaction temperature, reaction time, alkaline concentration, times of deacetylation, and time of ultrasonic degradation. Table S1 shows the preparation conditions and structural parameters of the chitosan with different DD and similar M_V_. Chitosan with ultra-high M_V_ were prepared by milder alkaline treatment from β-chitin or chitosan, even through multiple deacetylating in a short time [26]. The M_V_ of CTS-65 with the lowest DD was not obtained because of its low solubility in dilute acetic acid solution, and the CTS-70 was produced after two times’ milder ultrasonic degradation for slightly decreasing its M_V_. The CTS-90 with the highest DD was prepared from CTS-65 through three times’ deacetylation every for 1 h.

Table S2 shows the preparation conditions and structural parameters of chitosan with different M_V_ and similar DD. The CTS-1.3, which the M_V_ reached at 1.29 × 10^6^, was prepared from CTS-65 by milder alkaline deacetylation, and those chitosan with lower M_V_ were prepared from CTS-1.3 through ultrasonic degradation with different lengths of time. Elemental analysis results showed that ultrasonic degradation had little effect on the DD of chitosan [30], but the M_V_ of chitosan decreased with increasing ultrasonic time.

Table S3 shows the specifications of chitosan monofilaments as sutures. The diameter of chitosan monofilament slightly increased with the increase of DD and the decrease of M_V_ of the chitosan, possibly due to the slightly increased solubility of chitosan in the dilute acetic acid solution.

### 3.2. Appearance and Morphology of Monofilaments

**Figure 1:**
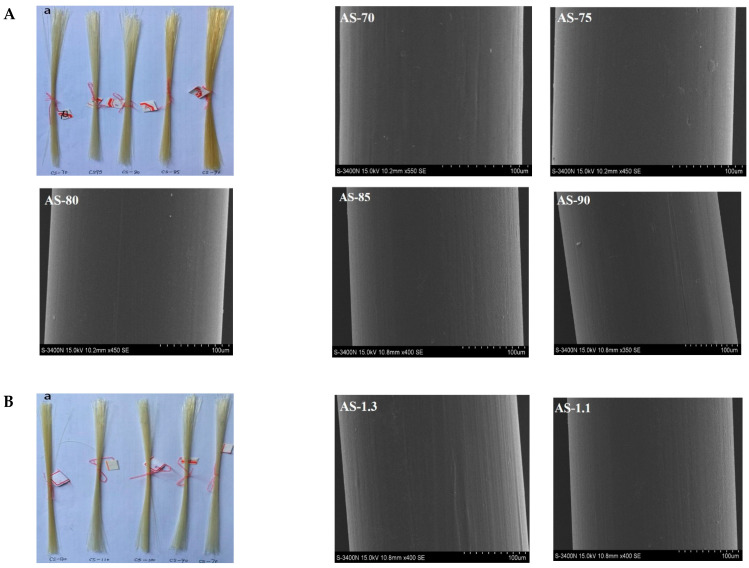

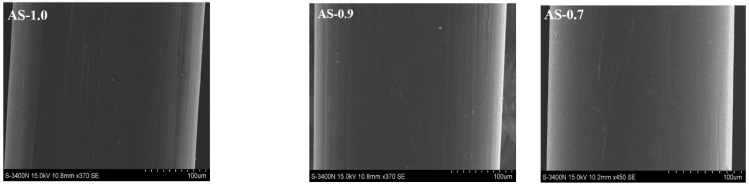
Appearance picture and surface SEM images of chitosan monofilaments with different DD (**A**) and chitosan monofilaments with different M_V_ (**B**).

Figure 1A shows the appearance picture and surface SEM images of chitosan monofilaments with different DD, and Figure 1B shows chitosan monofilaments with different M_V_. The appearance and morphology of chitosan monofilaments with different DD and M_V_ were uniform, and their pale-yellow surfaces were clean and free of stains. Their SEM images indicated that all surfaces look relatively smooth, with no air bubbles, pores, or depressions.

### 3.3. Mechanical Properties of Monofilaments

**Figure 2:**
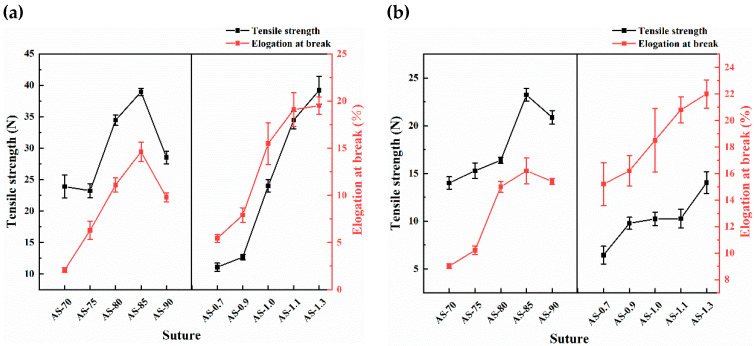
Mechanical properties of monofilaments (**a**) Dry and (**b**) Wet.

As a suture, chitosan monofilament should have excellent mechanical properties to hold wound tissue and withstand tensile forces. The experiment measured tensile strength and elongation at the break of dry and wet knotted monofilaments, as shown in Figure 2. The tensile strength and elongation at the break of the dry and wet knotted monofilament first increased and then decreased as the DD of chitosan increased, and the AS-85 prepared from the chitosan with (85.46 ± 1.13)% of DD and (1.16 ± 0.04) × 10^6^ of M_V_ exhibited the most optimal mechanical properties with 38.96 ± 0.56 N/23.24 ± 0.65 N of dry/wet tensile strength and (14.60 ± 1.03)%/(16.20 ± 0.98)% of dry/wet elongation at the break. It indicated that the monofilament prepared from the chitosan with medium-high DD and ultra-high M_V_ that was seized was of the highest toughness. As the DD increased, the molecular regulation of β-chitin was destroyed, and its crystallinity was lowered. The ductility and toughness of the knotted chitosan monofilament increased, so its tensile strength and elongation at the break increased. But the ultra-high DD (e.g., >85%) resulted in a more regular molecular arrangement and elevated crystallinity of chitosan, so the chitosan monofilament became more fragile and breakable [31,32]. Furthermore, the tensile strength and elongation at the break of the dry and wet knotted monofilament increased with the increasing of the M_V_ of the chitosan, which enhanced the intermolecular interaction including hydrogen bonding of the chitosan. When the chitosan monofilament is applied clinically as a surgical suture, it always appears in a wet state with lower tensile strength and higher elongation at the break than the dry.

### 3.4. Swelling Performance of Monofilaments

**Figure 3:**
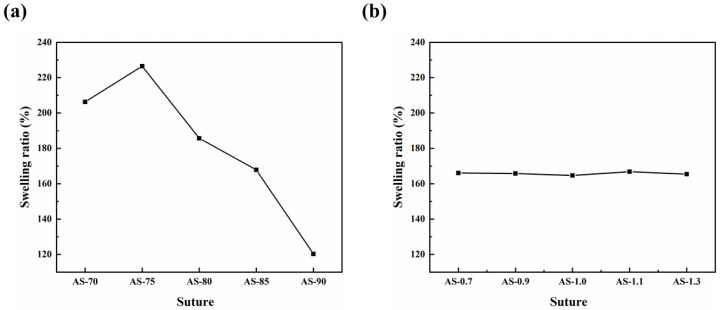
Effects of chitosan’s DD (**a**) and Mv (**b**) on swelling ratio of their monofilaments.

The results indicated that the swelling ratio of the chitosan monofilament decreased as the DD of the chitosan increased, due to the reduction of its acetamido group and the lowering of hydrophilicity, as shown in Figure 3. The slightly lower swelling ratio of AS-70 may have been derived from the lower water-solubility of CTS-70 (Tables S1 and S3). However, within the experimental range of M_V_, the M_V_ of the chitosan had no obvious effect on the swelling performance of the chitosan monofilament.

### 3.5. Ash Contents of Monofilaments

Theoretically, all chitosan monofilaments only contain chitosan with C/H/O/N elements. Hence, the inorganic impurities of chitosan and the contamination of alkaline coagulation bath should be controlled. The detected ash contents of all chitosan monofilaments, shown in Table 1, were less than 0.04% and met the requirements of the Chinese Pharmaceutical Industry Standards (YY 1116–2010).

### 3.6. In Vitro Enzymatic Degradation of Monofilaments

**Figure 4:**
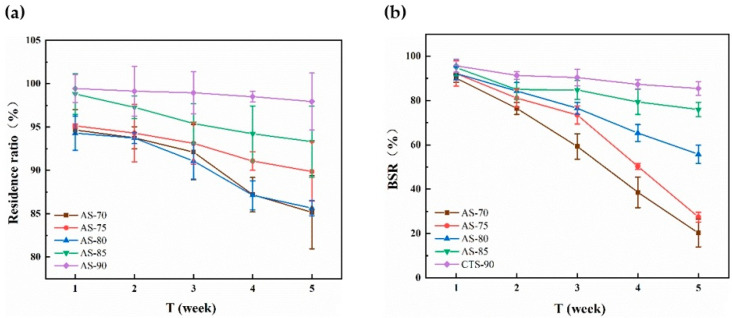
Effects of chitosan’s DD on residual ratio (**a**) and BSR (**b**) of its monofilament with enzymatic hydrolysis.

**Figure 5:**
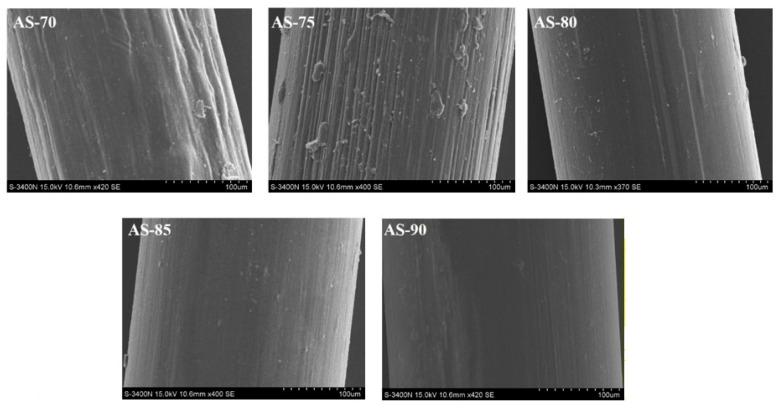
SEM images of chitosan (with different DD) monofilaments with enzymatic hydrolysis for five weeks.

The results indicated that the that the residual ratio and BSR of all chitosan monofilaments gradually decreased with increasing time, as shown in Figure 4. The lower DD of the chitosan resulted in the faster degradation of its monofilament, indicating that the degradation speed of chitosan and the number of acetamido groups of its molecular chain had a notably positive correlation, which was consistent with previous reports [33,34]. Among these chitosan monofilaments, AS-85 prepared from CTS-85 looked like an ideal surgical suture with moderate degradation speed and high BSR in 5 weeks. Furthermore, Figure 5 shows the SEM images of chitosan (with different DD) monofilaments with enzymatic hydrolysis for five weeks. With decreasing the DD of the chitosan, the surface of its monofilament bestrewed more depressions and pores, but AS-85 & AS-90 looked smoother.

**Figure 6:**
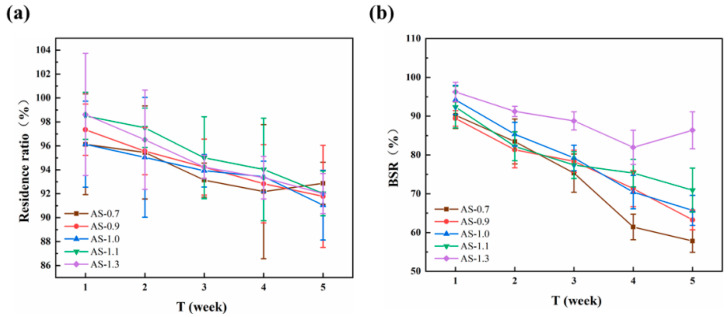
Effects of chitosan’s M_V_ on residual ratio (**a**) and BSR (**b**) of its monofilament with enzymatic hydrolys.

**Figure 7:**
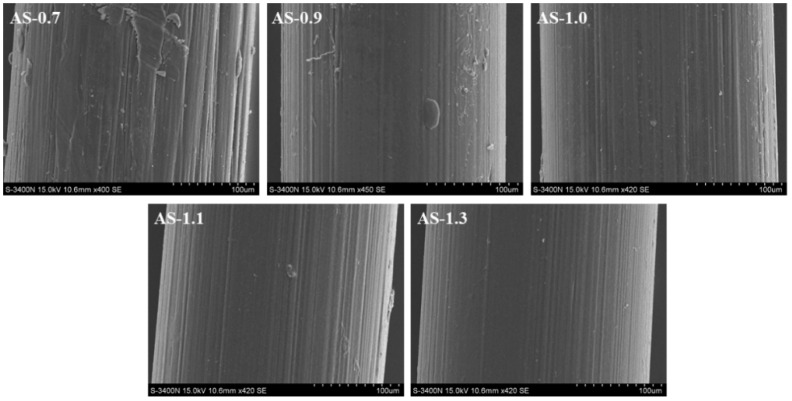
SEM images of chitosan (with different M_V_) monofilaments with enzymatic hydrolysis for five weeks.

The effects of the chitosan’s M_V_ on the residual ratio and BSR of its monofilament with enzymatic hydrolysis are shown in Figure 6. It looked as though the difference on the residual ratio for all monofilaments was not obvious, but the BSR of the monofilament decreased quickly with the decreasing of the M_V_ of the chitosan. After degradation for 5 weeks, the BSR of AS-1.1 & AS-1.3 were more than 70% and suitable as sutures. Figure 7 shows the SEM images of chitosan (with different M_V_) monofilaments with enzymatic hydrolysis for five weeks. With the decreasing of the M_V_ of the chitosan, the surface of its monofilament bestrewed more depressions and pores, but AS-1.0, AS-1.1, and AS-1.3 looked smoother.

### 3.7. In Vitro Cytotoxicity of AS-85

It was suggested that the excellent biocompatibility of the sutures is vital for their application in a clinical study. The in vitro cytotoxicity of the AS-85 suture was evaluated by an MTT assay using suture extracts on HFF-1 cells. As shown in Figure S1, the viability of the HFF-1 cells incubated for 1 d, 2 d, and 3 d in medium were all above 50% compared with the control, according to the ISO standard (ISO10993.12-2004), and the cytotoxicity of AS-85 was recorded as grade II, indicating that the absorbable surgical suture prepared in this experiment had slight cytotoxicity. This is likely because of the higher salt content in this suture.

## 4. Conclusions

In this study, the effects of the DD and M_V_ of chitosan on the properties of its monofilament were studied, including surface morphology, mechanical property, swelling ratio, ash content, in vitro enzymatic degradation, and in vitro cytotoxicity. According to the obtained results, AS-85 was chosen as the one that is most suitable as an absorbable surgical suture, which was spun from squid cartilage chitosan with DD~85% and M_V_~1.2 × 10^6^. Owing to the lower mechanical properties of the fibers spun from crab chitosan with low-moderate M_V_, chitosan has been absent from the applications of absorbable surgical sutures for a long time. However, the monofilament spun from chitosan with ultra-high M_V_ and appropriate DD exhibited outstanding mechanical properties and met the characteristic requirements as an absorbable surgical suture.

## Figures and Tables

**Table 1:** Ash contents of chitosan monofilaments.

Suture	Ash Content (%)	Suture	Ash Content (%)
AS-70	0.038	AS-1.3	0.036
AS-75	0.037	AS-1.1	0.037
AS-80	0.038	AS-1.0	0.039
AS-85	0.039	AS-0.9	0.037
AS-90	0.038	AS-0.7	0.038

## Data Availability

The data presented in this study are available on request from the corresponding author. The data are not publicly available due to privacy and ethics restrictions.

## Supplementary Materials

The following supporting information can be downloaded at: https://www.mdpi.com/article/10.3390/polym14071306/s1, Figure S1: Biocompatibility of AS-85 suture determined by MTT assay; Table S1: Preparation conditions and structural parameters of chitosan with different DD and similar M_V_; Table S2: preparation conditions and structural parameters of chitosan with different M_V_ and similar DD; Table S3: Specifications of chitosan monofilaments as sutures.
