# Supplementary material for: Effect of Squid Cartilage Chitosan Molecular Structure on the Properties of Its Monofilament as an Absorbable Surgical Suture
Source: Polymers (Basel). 2022 Mar 24;14(7):1306. doi: 10.3390/polym14071306 (PMC9003387; doi:10.3390/polym14071306)
Supplement: Supplementary file 1 [file polymers-14-01306-s001.zip › polymers-1505063-supplementary.pdf]

Supplementary materials

Table S1. Preparation conditions and structural parameters of chitosan with different DD and similar Mv.

Chitosn	Raw material	Alkaline deacetylation			Ultrasonic degradation		Structural parameters	
		Alkaline concentration (wt.%)	Time (h)	Temperature (°C)	Time (h)	Temperature (°C)	DD (%)	Mv (10^6)
CTS-65	β-chitin	30	2	70	-	-	64.30 ± 2.56	-
CTS-70	β-chitin	34	2	80	2	60	70.20 ± 1.14	1.20 ± 0.09
CTS-75	β-chitin	32	4	80	-	-	75.14 ± 0.87	1.18 ± 0.07
CTS-80	β-chitin	36	4	80	-	-	80.07 ± 0.65	1.15 ± 0.08
CTS-85	CTS-65	30	1	70	-	-	85.46 ± 1.13	1.16 ± 0.04
CTS-90	CTS-65	30	1 + 1 + 1	70	-	-	89.97 ± 1.02	1.10 ± 0.02

Table S2. preparation conditions and structural parameters of chitosan with different Mv and similar DD.

Chitosan	Raw material	Alkaline deacetylation			Ultrasonic degradation		Structural parameters	
		Alkaline concentration (wt.%)	Time (h)	Temperature (°C)	Time (h)	Temperature (°C)	DD (%)	Mv (10^6)
CTS-1.3	CTS-65	30	1	70	-	-	86.39 ± 0.71	1.29 ± 0.01
CTS-1.1	CTS-1.3	-	-	-	3	60	85.51 ± 0.98	1.07 ± 0.02
CTS-1.0	CTS-1.3	-	-	-	4	60	85.07 ± 1.04	0.98 ± 0.02
CTS-0.9	CTS-1.3	-	-	-	6	60	84.72 ± 0.65	0.89 ± 0.01
CTS-0.7	CTS-1.3	-	-	-	8	60	83.81 ± 0.58	0.74 ± 0.02

Table S3. Specifications of chitosan monofilaments as sutures.

Suture	Chitosan	Specifications			Suture	Chitosan	Specifications		
		Average diameter (mm)	Specification	Length (m)			Average diameter (mm)	Specification	Length (m)
AS-70	CTS-70	0.2320	4-0	2.0	AS-1.3	CTS-1.3	0.2312	4-0	2.0
AS-75	CTS-75	0.2411	4-0	2.0	AS-1.1	CTS-1.1	0.2486	4-0	2.0
AS-80	CTS-80	0.2612	4-0/T	2.5	AS-1.0	CTS-1.0	0.2719	4-0/T	2.5
AS-85	CTS-85	0.2817	4-0/T	2.5	AS-0.9	CTS-0.9	0.2872	4-0/T	2.5
AS-90	CTS-90	0.2938	4-0/T	2.5	AS-0.7	CTS-0.7	0.2964	4-0/T	2.5

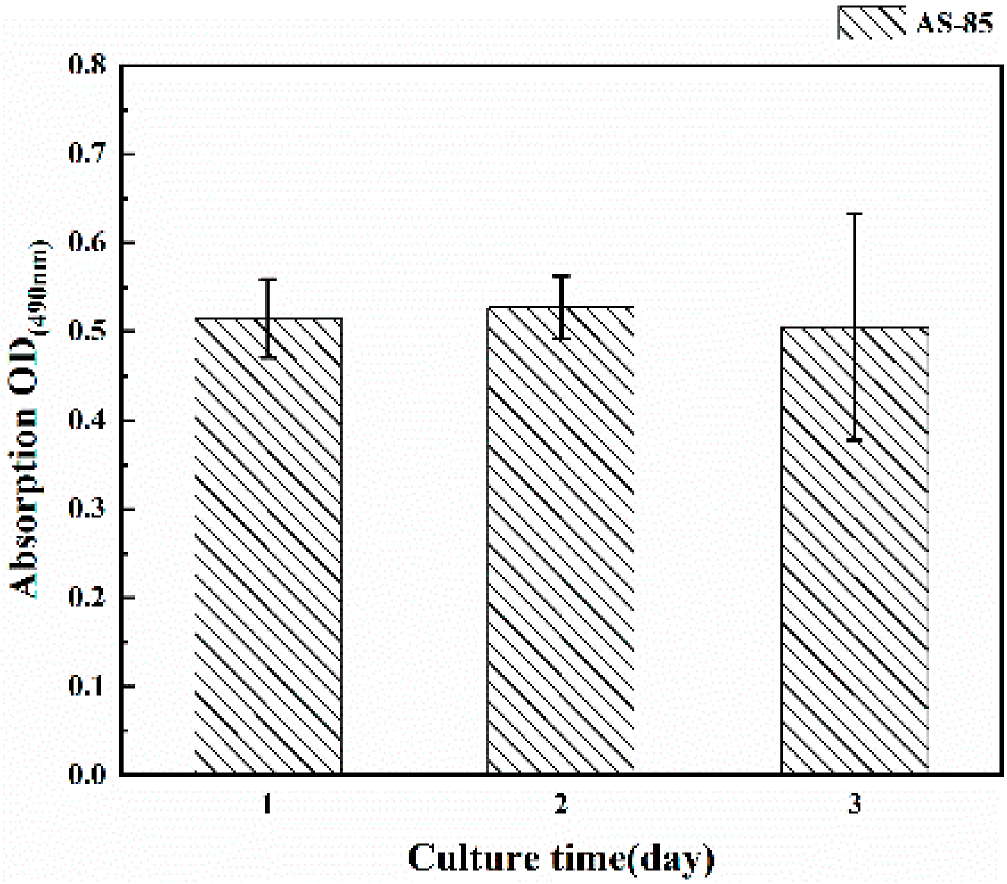

Figure S1. Biocompatibility of AS-85 suture determined by MTT assay.
